# A novel isoxazole compound CM2-II-173 inhibits the invasive phenotype of triple-negative breast cancer cells

**DOI:** 10.32604/or.2023.030411

**Published:** 2023-09-15

**Authors:** EUN SOOK KIM, SANGHEE KIM, AREE MOON

**Affiliations:** 1 Duksung Innovative Drug Center, College of Pharmacy, Duksung Women’s University, Seoul, 03169, Korea; 2 College of Pharmacy, Seoul National University, Seoul, 08826, Korea

**Keywords:** MMP-9, Cell invasion, Breast cells

## Abstract

Invasion and metastasis are important hallmarks of breast cancer and are the leading cause of patient mortality. Triple-negative breast cancer (TNBC) is an aggressive type of breast cancer characterized by a poor prognosis and a lack of effective targeted therapies. The present study investigated the inhibitory effect of a novel FTY720 derivative on the invasive phenotype of TNBC cells. Here, we showed that a novel compound with an isoxazole ring, 4-(3-Decylisoxazol-5-yl)-1-hydroxy-2-(hydroxymethyl)butan-2-aminium chloride (CM2-II-173), significantly inhibited invasiveness of MDA-MB-231 TNBC cells. Expression of matrix metalloproteinase (MMP)-9 and invasiveness of MCF10A normal breast cells induced by sphingosine-1-phosphate (S1P) were reduced by CM2-II-173 treatment. Activations of pMEK1, pAkt, pERK, and p38 MAPK by S1P were inhibited by treatment with CM2-II-173. Proliferation and anchorage-independent growth of MDA-MB-231 TNBC cells were significantly decreased by CM2-II-173. CM2-II-173 efficiently induced apoptosis in MDA-MB-231 TNBC cells. CM2-II-173 significantly inhibited invasive phenotypes of breast, liver, prostate, and ovarian cancer cells. CM2-II-173 exhibited a more potent effect on the invasiveness of MDA-MB-231 TNBC cells compared to FTY720. Taken together, this study demonstrated that CM2-II-173 has the potential to be a lead compound that can inhibit cancer progression of not only TNBC cells, but also of liver, prostate, and ovarian cancer cells.

## Introduction

Breast cancer is the most frequently diagnosed cancer worldwide and is the leading cause of cancer mortality among females [1]. Cancer invasion and metastasis are major hallmarks of breast cancer and are the leading cause of patient mortality [2]. Triple-negative breast cancer (TNBC), which has no receptors for estrogen, HER2, and progesterone, is a complex disease with a poor prognosis because of its high recurrence rate and high malignancy [3]. Currently, there are no effective targeted therapies, which results in poor survival compared to other subtypes of breast cancer [4].

Inflammation influences breast cancer progression and metastasis by modulating the microenvironment around cancers [5]. An inflammatory lipid sphingosine-1-phosphate (S1P) is involved in the regulation of various biological processes, including cell growth, survival, and invasion. Matrix metalloproteinases (MMPs) play significant roles in tumor invasion and migration. High expressions of MMP-2 and MMP-9 are associated with enhanced invasion in breast cells [6–8]. Recently, we revealed that S1P induced an invasive phenotype of human mammary epithelial cells through MMP-9 upregulation [9,10]. S1P was important for aggressiveness of TNBC cells [10]. Blocking the S1P signaling pathway can be a useful strategy to inhibit malignant transformation of breast cancer.

FTY720, a known S1P receptor antagonist [11,12], inhibited metastasis in mouse breast cancer and prostate cancer models [13,14]. We have previously shown that FTY720 inhibited the S1P-mediated induction of MMP-9 and invasion of MCF10A human breast epithelial cells [10]. However, FTY720 showed no significant inhibitory effect on invasiveness of MDA-MB-231 TNBC cells. In the present study, we synthesized FTY720-like sphingolipids and examined their inhibitory effects on the invasiveness of TNBC cells. Here, we identified a novel isoxazole compound CM2-II-173, which inhibited the invasiveness of TNBC cells as well as of liver, liver, prostate, and ovarian cancer cell lines.

## Materials and Methods

### Reagents

S1P was obtained from Sigma-Aldrich (St Louis, MO, USA). FTY720 was obtained from Selleck Chemicals (Houston, TX, USA). Primary antibodies for phospho-p38, p38, phospho-ERK1/2, ERK1/2, phospho-Akt, Akt, phospho-MEK1/2, MEK1/2, PARP, and β-actin were obtained from Cell Signaling Technology (Beverly, MA, USA). MMP-2 antibody was obtained from R&D Systems (Minneapolis, MN, USA). MMP-9, Bcl2, Caspase-3, and Bax antibodies were obtained from Santa Cruz Biotechnology, Inc. (Santa Cruz, CA, USA).

### Chemistry

Spectral and Analytical Data of CM2-II-173. 1H NMR (800 MHz, CD3OD) δ 6.11 (s, 1H), 3.65 (dd, J = 11.6, 15.4 Hz, 4H), 2.86 (td, J = 4.3, 8.2 Hz, 2H), 2.61 (t, J = 7.6 Hz, 2H), 2.08–2.06 (m, 2H), 1.64 (td, J = 7.4, 14.7 Hz, 2H), 1.33–1.31 (m, 4H), 1.30–1.26 (m, 10H), 0.89 (t, J = 7.1 Hz, 3H); 13C NMR (200 MHz, CD3OD) δ 174.1, 166.6, 102.8, 63.1, 62.5, 33.9, 31.5, 31.4, 31.23, 31.18, 31.05, 30.99, 30.1, 27.6, 24.5, 22.3, 15.2; IR (neat) υmax = 2919, 2853, 2407, 1610, 1459, 1060, 995, 754, 723 (cm^–1^); HRMS (FAB) calcd. for C_17_H_35_N_2_O_3_ [M−Cl−]+ 327.2648, found 327.2657.

### Cell lines

MCF10A cells were cultured and maintained as described in the previous document [15]. MDA-MB-231 and Hs578T TNBC cells were obtained from the Korean Cell Line Bank. Hepatocellular carcinoma cell lines (SNU387 and SK-Hep-1) were kindly provided by Prof. S.G. Kim (Dongguk University, Seoul, Korea). Prostate (DU145) and ovarian (SKOV3) cancer cells were cultured as previously described [16].

### Gelatin zymogram assay

The gelatin degradation activity in the conditioned medium (CM) was assessed using the gelatin zymogram assay [17]. The CM was collected and subjected to centrifugation at 10,000 g for 5 min to eliminate cell debris. The protein concentration in the CM was determined using BCA protein assay reagents (Pierce, Rockford, IL, USA). Equal amounts of CM were mixed with Laemmli SDS sample buffer, incubated for 15 min at room temperature, and then electrophoresed. After electrophoresis, the gels were washed in renaturing buffer and incubated overnight at 37°C. The gels were stained with staining buffer and then subjected to destaining. Clear bands indicating gelatinase activity were observed against the blue-stained gelatin background.

### In vitro invasion assay

*In vitro* invasion assays [17] were performed using a 24-Transwell plate with a polyethylene terephthalate membrane (BD, Franklin Lakes, NY, USA). Cells (2 × 104 cells/well) were seeded onto the insert of a transwell chamber. After incubating the cells at 37°C for 24 h in a humidified atmosphere with 5% CO_2_. Following incubation, the invaded cells that had adhered to the membrane were stained with 0.5% crystal violet for 20 min at room temperature. To obtain quantitative measurements, the membrane filter stained with crystal violet was carefully excised and eluted with 30% acetic acid for 5 min. The absorbance of the eluate was measured using a spectrophotometer at a wavelength of 595 nm.

### Immunoblot analysis

Whole cell lysates were prepared using SDS lysis buffer. Equal amounts of protein extracts were subjected to SDS-PAGE analysis, followed by electrophoretic transfer to a nitrocellulose membrane. The immunoreactive proteins were detected using a WesternBright ECL kit (cat no K-12045-D20, Advansta Inc., Menlo Park, CA, USA). Immunoreactive proteins were visualized by exposing the membranes to UV light using a FluorChem E documentation system (ProteinSimple, Santa Clara, CA, USA). The protein bands were quantified using AlphaView software (ProteinSimple) for quantification.

### Soft agar assay

Anchorage-independent growth was evaluated in MDA-MB-231 cells treated with CM2-II-173. Briefly, 1 ml RPMI medium (1.5×) containing 0.8% agarose was added to culture dishes and allowed to solidify to form the base agar layer. Next, cells were mixed with 0.8 ml RPMI medium containing 0.3% agarose and added on top of the base agar layer. The cells were then incubated and cultured for 14 days to allow for colony formation and assessment of anchorage-independent growth. Colonies were visualized with a microscope. Original magnification: ×100.

### Proliferation assay

Crystal violet staining was used to detect cell proliferation [18]. Briefly, cells were seeded into individual wells of 96 well plates and incubated overnight. CM2-II-173 was applied for 24 h at different concentrations. Cells were then stained with a 0.2% crystal violet solution for 20 min and washed three times using PBS. Stained cells were dissolved with 1% SDS, and the optical density was measured at 540 nm using an ELISA reader (Synergy 2; BioTek Instruments, Inc., Winooski, VT, USA).

### FACS analysis

The cells were grown in 100 mm plates and incubated for 24 h with 2 μM CM2-II-173. Following the incubation period, the cells were harvested and washed twice with phosphate-buffered saline. Subsequently, the cells (5 × 10^5^) were stained with FITC-conjugated annexin V and propidium iodide for 15 min at room temperature in 1× binding buffer. The stained cells were then analyzed using a Novocyte Flow Cytometer (ACEA Biosciences Inc., CA, USA).

### Cell viability assay

The cell viability analysis was performed using the Cell Counting Kit-8 (CCK-8; (Dojindo Laboratories, Inc., Japan)). The cells were seeded in 96‑well plates (2 × 104 cells/well) and incubated for 24 h with various concentrations of CM2-II-173. After adding 10 μl of CCK-8 solution to the cells, they were incubated for 1–4 h. The absorbance was measured at 450 nm using a microplate reader.

### Statistical analysis

Statistical significance was analyzed by ANOVA using GraphPad Prism 6 (GraphPad Software, Inc., La Jolla, CA, USA). Multi‑comparison was performed using Dunnett’s multiple comparisons test. The data are shown as the mean ± SD from three independent experiments. *p* ≤ 0.05 was considered statistically significant.

## Results

### CM2-II-173 inhibits invasiveness of MDA-MB-231 TNBC cells

**Figure 1:**
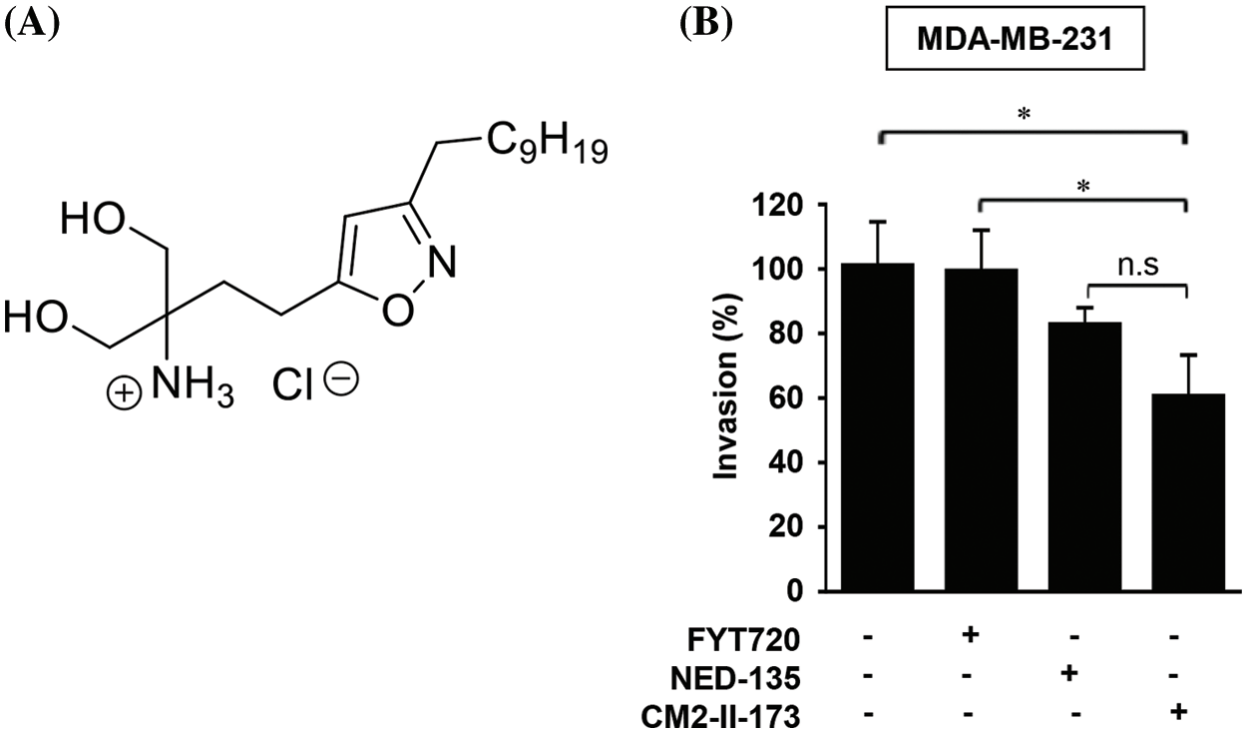
CM2-II-173 inhibits invasiveness of MDA-MB-231 TNBC cells. (A) Structure of the CM2-II-173 compound. (B) MDA-MB-231 TNBC cells were treated with 2 μM FTY720, NED-135, or CM2-II-173 for 24 h. The invasion ability (%) of the cells was calculated by comparing the invasive capacity of the control group, which was considered as 100% (one‑way ANOVA, *p < 0.05).

To identify novel compound(s) that can effectively inhibit invasion of TNBC cells, we synthesized an FTY720-like sphingolipid analog containing a heteroaromatic ring instead of a phenyl ring of FTY720. The chemical structure of CM2-II-173, 4-(3-Decylisoxazol-5-yl)-1-hydroxy-2-(hydroxymethyl)butan-2-aminium chloride, containing an isoxazole ring is depicted in Fig. 1A. As shown in Fig. 1B, CM2-II-173 exhibited a more potent effect on the invasiveness of MDA-MB-231 TNBC cells compared to FTY720 and NED-135.

Consistent with our previous report [19], FTY720 did not significantly inhibit the invasive capacity of MDA-MB-231 TNBC cells. Another antagonist of S1P receptor, NED-135 [19], also inhibited invasion of cells, but to a lesser extent than CM2-II-173. These results demonstrated that CM2-II-173 may exert a more effective inhibition on TNBC cell invasiveness, compared to FTY720 and NED-135.

### CM2-II-173 inhibits the S1P-induced invasive phenotype and MMP-9 expression in MCF10A cells

**Figure 2:**
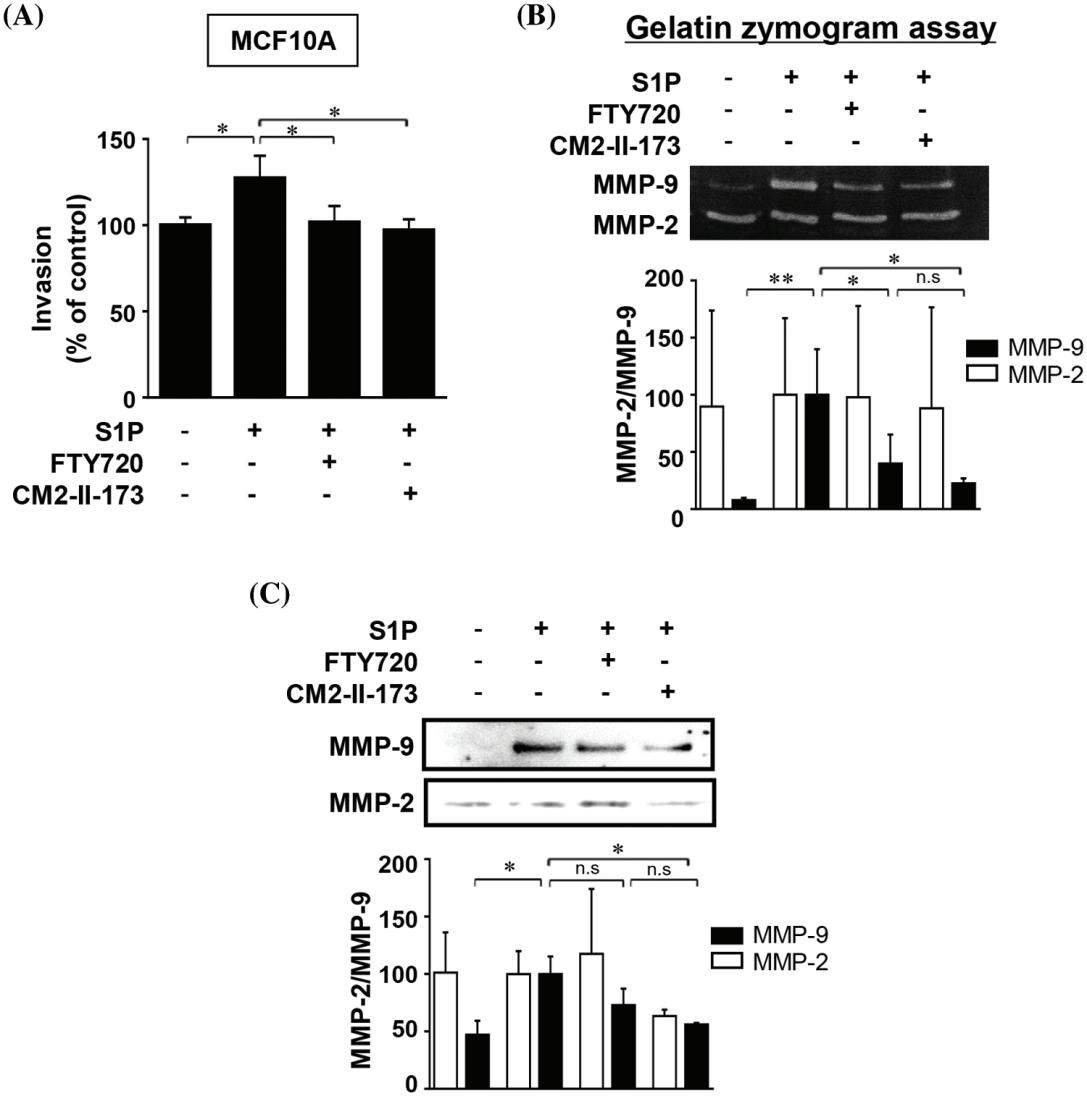
CM2-II-173 inhibits the S1P-induced invasive phenotype and MMP-9 expression in MCF10A cells. (A) Invasion assay was performed on MCF10A cells treated with 10 μM S1P and 2 μM FTY720 or CM2-II-173 for 24 h (one‑way ANOVA, *p < 0.05). (B and C) Gelatin zymogram assay and immunoblot analysis (MMP-2: 72 kDa, MMP-9: 92 kDa) were conducted on conditioned media from cells (one‑way ANOVA, *p < 0.05 and **p < 0.01).

To determine whether CM2-II-173 inhibits S1P-induced cell invasion in MCF10A cells, an *in vitro* invasion assay was conducted. Treatment of cells with CM2-II-173 markedly inhibited the S1P-induced invasiveness in MCF10A breast cells (Fig. 2A). We next investigated the effect of CM2-II-173 on MMP-9 expression induced by S1P. Treatment of 2 μM CM2-II-173 more effectively inhibited S1P-induced MMP-9 expression in MCF10A cells compared to FTY720, as shown by the gelatin zymogram assay and immunoblot analysis (Figs. 2B and 2C, respectively). When the toxicity assessment of CM2-II-173 in MCF10A cells was performed, CM2-II-173 did not affect the cell viability at a concentration of 2 μM (data not shown).

### CM2-II-173 suppresses S1P-induced activation of signaling molecules

**Figure 3:**
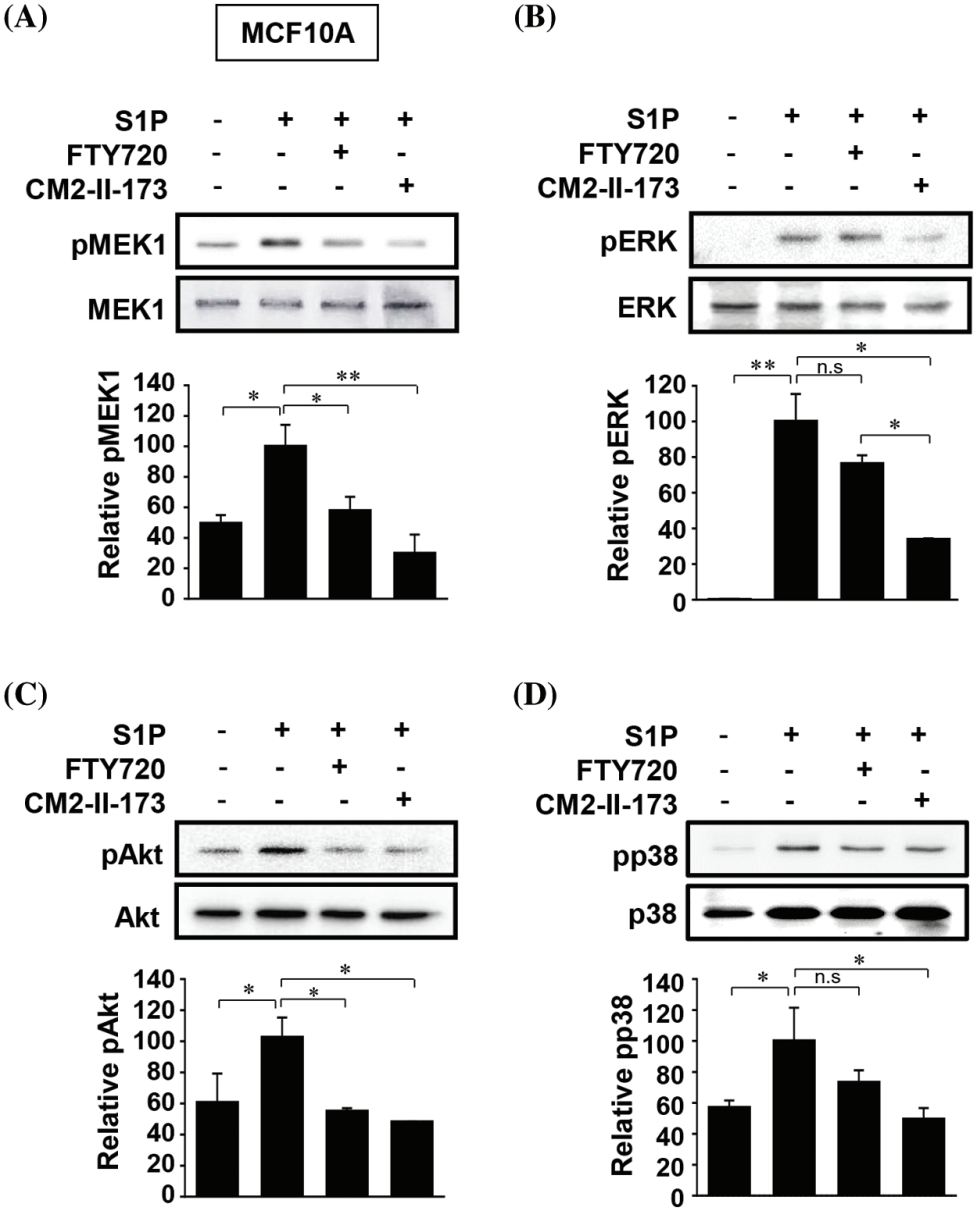
CM2-II-173 suppresses S1P-induced activation of signaling molecules. MCF10A cells were treated with 10 μM S1P and 2 μM FTY720 or 2 μM CM2-II-173 for 24 h. (A–D) Immunoblot analysis was performed to assess the levels of phosphorylated MEK1 (45 kDa), ERK (42 kDa), Akt (60 kDa), and p38 MAPK (38 kDa) (one‑way ANOVA, *p < 0.05 and **p < 0.01).

S1P induced activation of MEK, ERKs, Akt, and p38 MAPKs in MCF10A cells, which play an important role in S1P-induced upregulation of MMP-9 and invasion [9]. We investigated the effect of CM2-II-173 on the activation of these signaling molecules. Treatment of 2 μM CM2-II-173 more effectively inhibited the phosphorylation of MEK, ERKs, Akt, and p38 MAPK induced by S1P compared to FTY720 (Figs. 3A–3D, respectively). These data demonstrate that CM2-II-173 was an effective antagonist of the S1P receptor; and thus, it inhibited the S1P-triggered signaling pathways. The results suggested a possible involvement of MEK, ERKs, Akt, and p38 MAPK in the inhibitory effect of CM2-II-173 on invasion.

### CM2-II-173 inhibits proliferation and anchorage-independent growth of MDA-MB-231 TNBC cells

**Figure 4:**
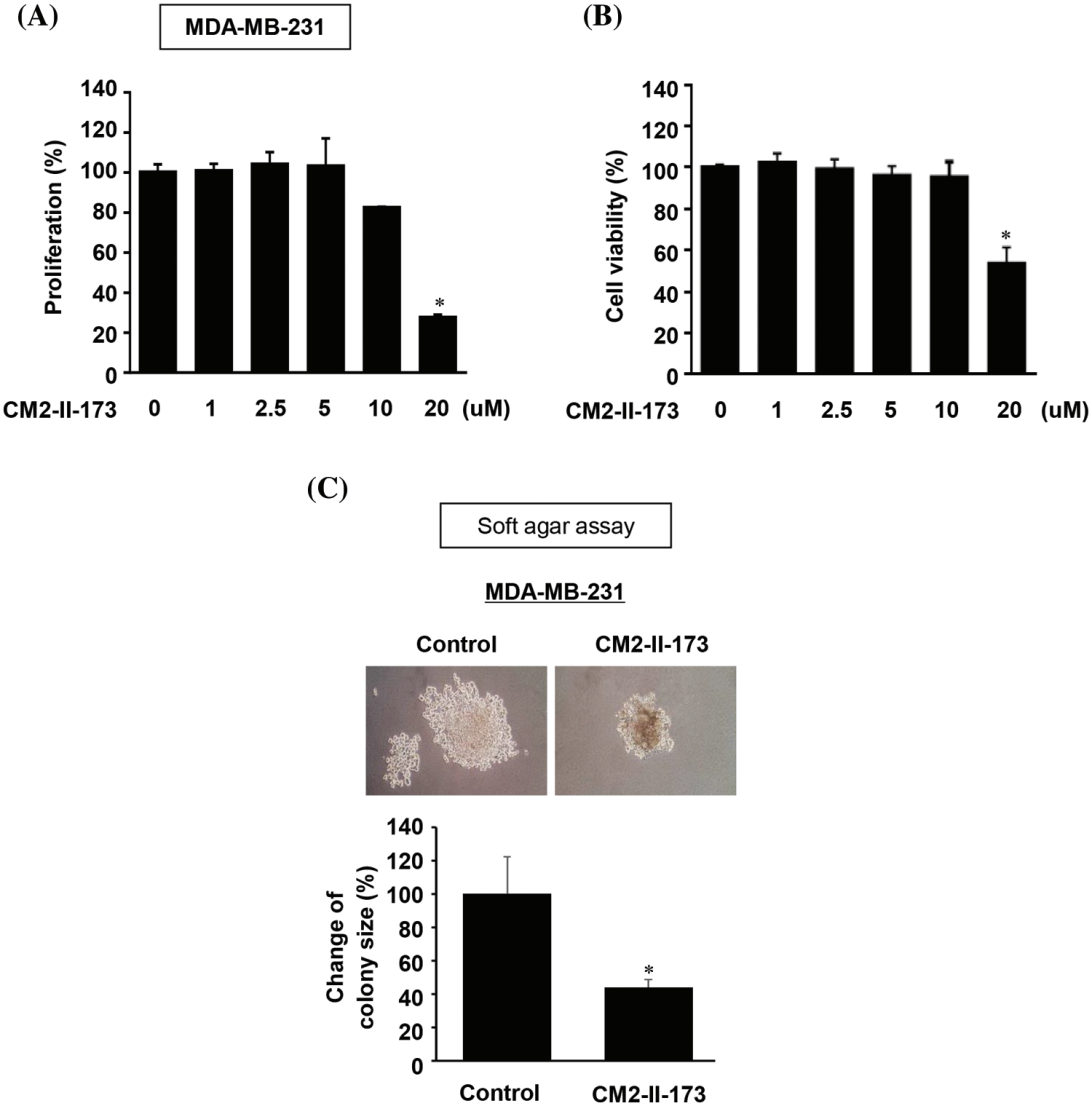
CM2-II-173 inhibits proliferation and anchorage-independent growth of MDA-MB-231 TNBC cells. (A) Cell proliferation was assessed by crystal violet staining in MDA-MB-231 TNBC cells treated with different concentrations of CM2-II-173 (one‑way ANOVA, *p < 0.05). (B) Cell viability was measured in MDA-MB-231 TNBC cells treated with different concentrations of CM2-II-173 (one‑way ANOVA, *p < 0.05). (C) Anchorage-independent growth was measured in MDA-MB-231 TNBC cells treated with 2 μM CM2-II-173 (one‑way ANOVA, *p < 0.05). Original magnification: ×100.

To investigate the effect of CM2-II-173 on cell proliferation, MDA-MB-231 TNBC cells were treated with various concentrations of CM2-II-173. As shown in Fig. 4A, treatment with CM2-II-173 for at various concentrations 24 h significantly inhibited the proliferation of MDA-MB-231 TNBC cells at a concentration of 20 μM. The cell viability of MDA-MB-231 TNBC cells was significantly reduced by treatment with 20 μM CM2-II-173 (Fig. 4B). Anchorage-independent growth has been extensively investigated as a key characteristic of malignant cancer cells [20]. We next investigated whether CM2-II-173 affects the anchorage-independent growth of MDA-MB-231 TNBC cells. CM2-II-173 inhibited the growth of MDA-MB-231 TNBC cells on soft agar (Fig. 4C). Taken together, the data demonstrated that treatment of TNBC cells with CM2-II-173 significantly inhibited the proliferation and anchorage-independent growth.

### CM2-II-173 induces apoptosis in MDA-MB-231 TNBC cells

**Figure 5:**
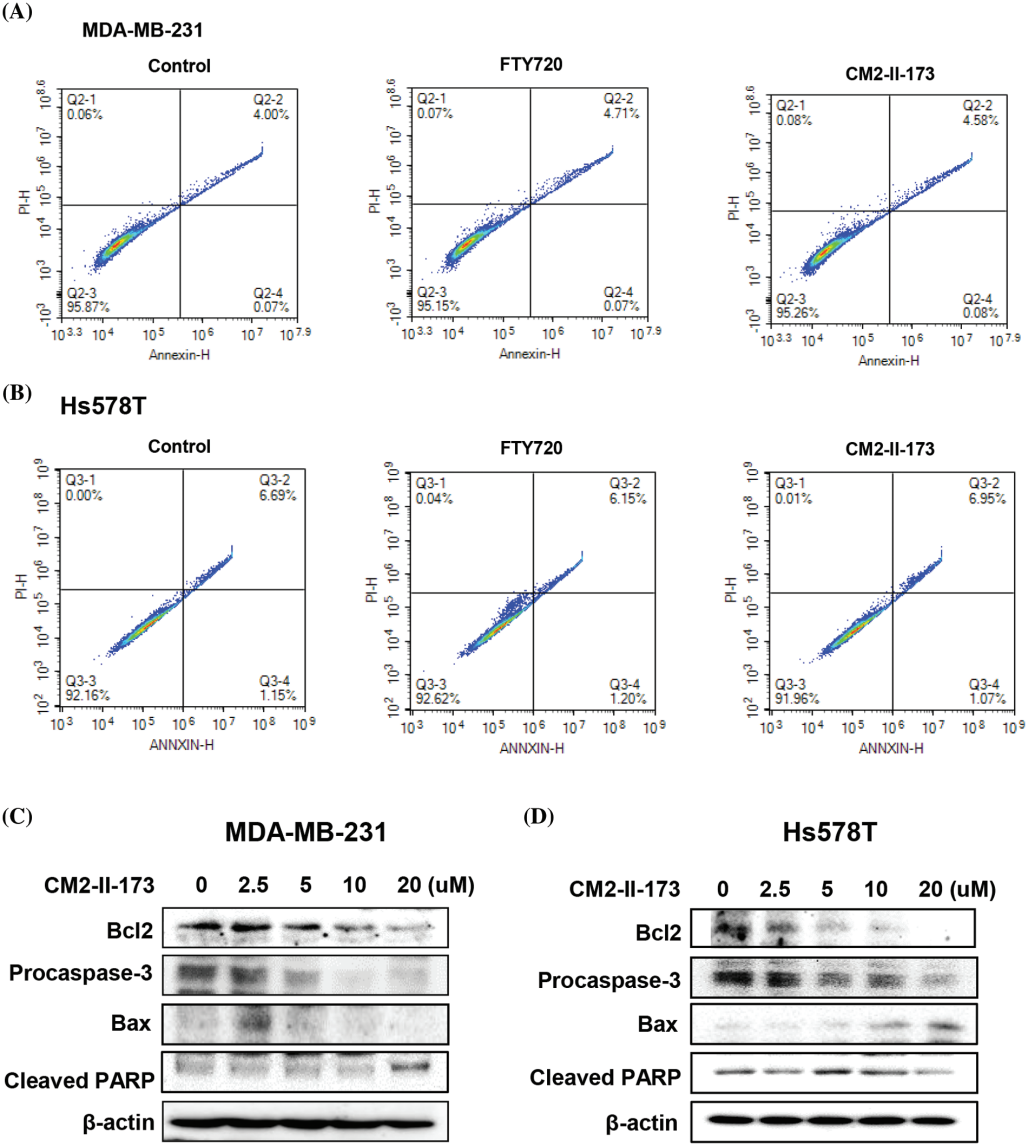
CM2-II-173 induces apoptosis in MDA-MB-231 and Hs578T TNBC cells. (A and B) FACS analysis was performed on cells treated with 2 μM CM2-II-173 or FTY720 for 24 h. (C and D) Cells were treated with CM2-II-173 at the concentrations indicated. Immunoblot analyses were performed to determine the levels of Bcl-2, pro-caspase-3, Bax, and cleaved PARP.

To examine whether CM2-II-173 affects apoptosis in MDA-MB-231 and Hs578T TNBC cells, FACS analysis was conducted. Treatment with 2 μM CM2-II-173 or FTY720 led a slight increase in the population of annexin-positive cells and annexin/PI-positive cells compared to the control cells (Fig. 5A). In Hs578T cells, FTY720 and CM2-II-173 showed a similar level of cell apoptosis compared to the control (Fig. 5B).

To evaluate the molecular mechanisms underlying the CM2-II-173-induced apoptosis of MDA-MB-231 and Hs578T TNBC cells, we determined the protein levels of apoptosis-related molecules. Expression of the anti-apoptotic protein Bcl-2 was decreased in a dose-dependent manner, whereas the expression of death-promoting protein Bax was increased by treatment with 2.5 uM CM2-II-173 (Figs. 5C and 5D). The level of pro-caspase-3 was markedly decreased by CM2-II-173 treatment, suggesting that CM2-II-173 induced the activation of caspase-3 in MDA-MB-231 TNBC cells. PARP-1 is known to be involved in apoptosis, and its cleavage has been suggested as a marker of caspase-dependent apoptosis [21]. Treatment with CM2-II-173 induced cleavage of PARP, indicating that CM2-II-173 activated the apoptotic pathway. These results suggested that CM2-II-173 induced apoptosis by downregulating Bcl-2 and upregulating Bax in TNBC cells.

### CM2-II-173 inhibits the invasiveness of cancer cells

**Figure 6:**
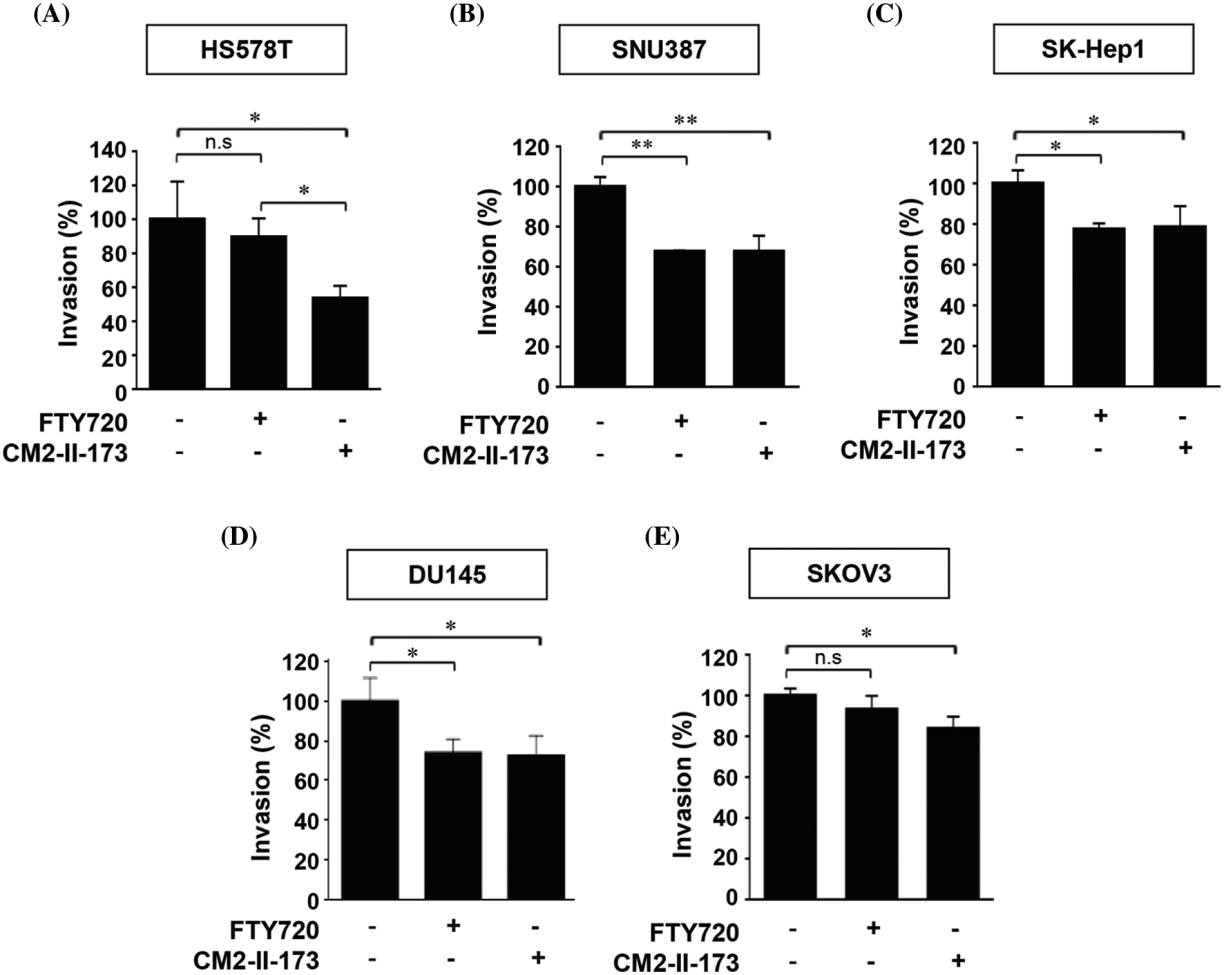
CM2-II-173 inhibits the invasiveness of cancer cells. (A–E) *In vitro* invasion assay was conducted on breast, liver, prostate, and ovarian cancer cells treated with 2 μM CM2-II-173 or FTY720 for 24 h. The invasion ability (%) was calculated by comparing the invasiveness of cells treated with the control as 100% (one‑way ANOVA, *p < 0.05 and **p < 0.01, respectively).

We next examined the inhibitory effect of CM2-II-173 and FTY720 on invasive phenotypes of TNBC cells and several cancer cell lines. Treatment with 2 uM CM2-II-173 showed a stronger inhibition of cell invasion in Hs578T cells compared to FTY720 (Fig. 6A). Prominent decreases in the invasive phenotypes were observed in liver cancer cell lines SNU387 and SK-Hep1 upon treatment with CM2-II-173 (Figs. 6B and 6C). Treatment with CM2-II-173 significantly inhibited the invasion of DU145 prostate cancer cells and SKOV3 ovarian cancer cells (Figs. 6D and 6E). The inhibitory effect of CM2-II-173 on cell invasion was more potent in Hs578T TNBC and SKOV3 cells compared to FTY720. The results suggested a potential application of CM2-II-173 for the development of anti-invasive agents for breast, liver, prostate, and ovarian cancers. Taken together, we demonstrated that our novel compound CM2-II-173 exerted inhibitory effects on various cancers, including TNBC, liver, prostate, and ovarian cancers.

## Discussion

Tumor cell invasion is an early step in the metastatic cascade that marks the beginning of a transition from benign to malignant progression [22]. TNBC is one of the highly aggressive cancers with poor clinical outcomes [23]. An inflammatory lipid S1P has been involved in various diseases, including cancer, atherosclerosis, and kidney disease [24–27]. Therefore, targeting S1P signaling may be a promising strategy to block cancer growth and progression. Various S1P modulators have been developed, but the effect of these compounds in cancer models remains to be investigated [28].

In an effort to develop a novel compound with an inhibitory effect on TNBC cell invasion, we synthesized derivatives of FTY720, a known antagonist of S1P. Here, we showed that CM2-II-173 compound with an isoxazole ring inhibited the invasion of MDA-MB-231 TNBC cells. FTY720 failed to significantly inhibit the invasive capacity of these cells. CM2-II-173 contained a heteroaromatic ring, instead of a phenyl ring of FTY720, suggesting the isoxazole ring as an important structure for the anti-invasive effect. In addition, we showed that CM2-II-173 exerted a stronger inhibitory effect than another S1P antagonist NED-135 [19] on TNBC cells.

Unlike FTY720 with a phenyl ring, CM2-II-173 contained a heteroaromatic ring, isoxazole. Derivatives containing isoxazole exerted anticancer [29–31] and anti-inflammatory activities [32,33]. Diarylisoxazole derivatives exhibited anti-cancer activity against MDA-MB-453 cells, a model of the luminal androgen receptor subtype of TNBC [34]. Combination of the trimethoxyphenyl moiety with isoxazole showed cytotoxic activity against HeLa, MCF-7, and HCT116 cells [35,36]. Taken together, the isoxazole moiety could be used as a key structure in the design of novel anti-cancer drugs. Further studies are needed to elucidate the structure-activity relationship of S1P antagonists and anti-invasive effect on TNBC cells.

Activation of S1P receptors by S1P regulates signaling pathways involving AKT and ERK1/2 through G-protein activity [9]. In order to treat malignant cancers, such as TNBC, it is necessary to block the signaling pathways that lead to metastasis. We demonstrated that CM2-II-173 suppressed the active phosphorylated forms of ERK1/2, p38, and AKT, which were important signals for induction of invasion and MMP-9 by S1P.

Since evasion of apoptosis is a key feature of cancer, use of cell death mechanisms in cancer treatment is a highly effective strategy [37]. Our data showed that CM2-II-173 induced apoptosis in TNBC cells with a significant decrease in Bcl-2, a representative apoptosis suppressor gene. Pro-caspase-3 was decreased by CM2-II-173, which induced the cleavage of PARP, a DNA recovery enzyme [38]. PARP1 cleavage is observed in the early and intermediate stages of apoptosis [39]. During apoptosis, the activation of PARP induces the poly(ADP-ribosyl)ation of important nuclear proteins required for the progression of apoptosis, while it is cleaved and inactivated by caspase-3 [40]. PARP inhibitors have shown promising clinical outcomes for breast cancer types, including TNBC [41]. These results indicate that CM2-II-173 induced apoptosis by downregulating Bcl-2 and upregulating Bax in breast cancer cells.

Recently, various S1P receptor-targeting S1P modulators have been developed, and some of them have been shown to play important roles in cancer. For example, FTY720, an S1P modulator that binds to S1PR1, S1PR3, S1PR4, and S1PR5, has been shown to inhibit tumor growth and aggressiveness in various cancer models. However, other S1P modulators have not been extensively studied or tested in cancer models [28]. Therefore, there is a need for the development of new compounds that can inhibit the invasive ability of breast cancer cells.

The present study demonstrated that CM2-II-173 effectively inhibited the invasiveness of TNBC cells compared to FTY720. We present the potential of CM2-II-173 as a therapeutic agent that can prevent cancer metastasis and block the S1P-mediated cancer progression.

## Data Availability

All data are available from the corresponding author upon request.
